# The Evolving Classification of Meningiomas: Integration of Molecular Discoveries to Inform Patient Care

**DOI:** 10.3390/cancers16091753

**Published:** 2024-04-30

**Authors:** S. Joy Trybula, Mark W. Youngblood, Constantine L. Karras, Nikhil K. Murthy, Amy B. Heimberger, Rimas V. Lukas, Sean Sachdev, John A. Kalapurakal, James P. Chandler, Daniel J. Brat, Craig M. Horbinski, Stephen T. Magill

**Affiliations:** 1 Department of Neurological Surgery, Northwestern University Feinberg School of Medicine, Chicago, IL 60611, USA; 2 Department of Neurology, Northwestern University Feinberg School of Medicine, Chicago, IL 60611, USA; 3 Department of Radiation Oncology, Northwestern University Feinberg School of Medicine, Chicago, IL 60611, USA; 4 Department of Pathology, Northwestern University Feinberg School of Medicine, Chicago, IL 60611, USA

**Keywords:** meningioma, molecular, methylation, WHO grade, copy number variant, gene expression panel, history

## Abstract

**Simple Summary:**

Meningiomas are the most common intracranial tumors, and significant advances have been made in our understanding of the biology that leads to meningioma growth and aggressiveness. This review summarizes molecular advances and the historical contexts that led to them.

**Abstract:**

Meningioma classification and treatment have evolved over the past eight decades. Since Bailey, Cushing, and Eisenhart’s description of meningiomas in the 1920s and 1930s, there have been continual advances in clinical stratification by histopathology, radiography and, most recently, molecular profiling, to improve prognostication and predict response to therapy. Precise and accurate classification is essential to optimizing management for patients with meningioma, which involves surveillance imaging, surgery, primary or adjuvant radiotherapy, and consideration for clinical trials. Currently, the World Health Organization (WHO) grade, extent of resection (EOR), and patient characteristics are used to guide management. While these have demonstrated reliability, a substantial number of seemingly benign lesions recur, suggesting opportunities for improvement of risk stratification. Furthermore, the role of adjuvant radiotherapy for grade 1 and 2 meningioma remains controversial. Over the last decade, numerous studies investigating the molecular drivers of clinical aggressiveness have been reported, with the identification of molecular markers that carry clinical implications as well as biomarkers of radiotherapy response. Here, we review the historical context of current practices, highlight recent molecular discoveries, and discuss the challenges of translating these findings into clinical practice.

## 1. Introduction

**Figure 1:**
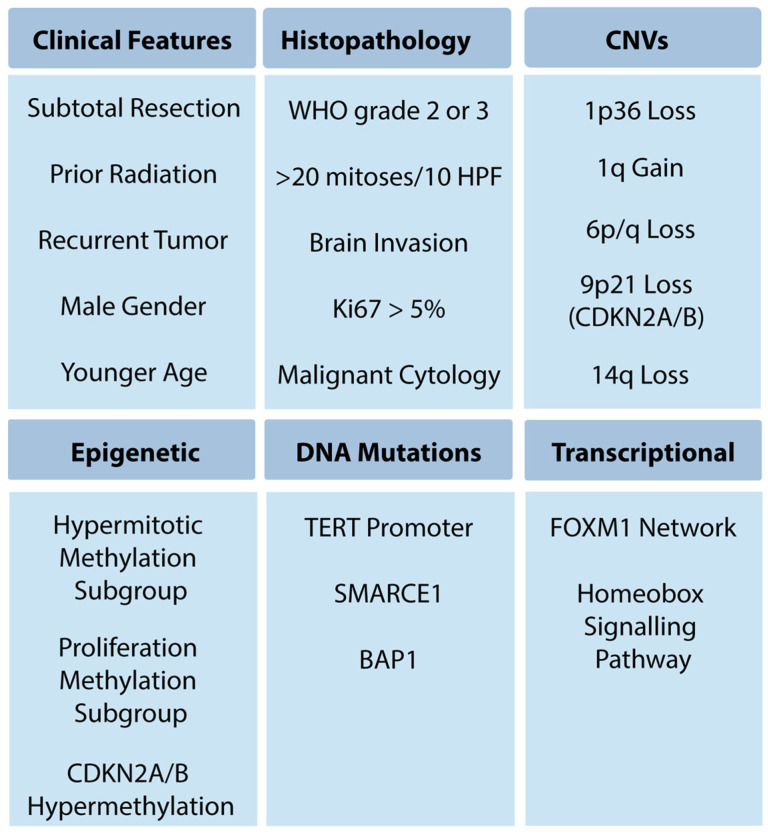
High-risk meningioma features.

**Figure 2:**
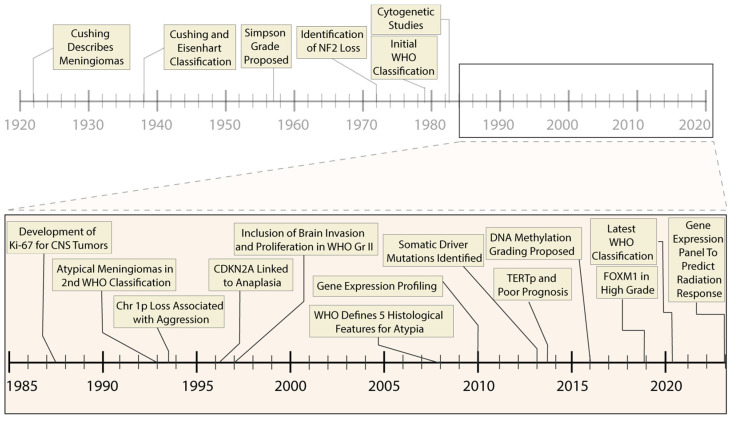
**Timeline of prognostic advances in meningiomas**. Progressive molecular and histological characterization has led to refinement in the clinical treatment of meningiomas, including improvement in prognostic accuracy and selection of appropriate therapeutic interventions. The past thirty-five years have been marked by rapid molecular advances (inset panel), including high-throughput sequencing and genome-wide epigenetic studies.

Meningioma is the most common primary central nervous system tumor, with over 37,000 cases per year in the United States, greater than the 16,000 gliomas of all grades per year [1]. While most meningiomas are sporadic, they can occur in genetic syndromes, including neurofibromatosis type 2, BAP1 tumor predisposition syndrome, and Gorlin syndrome [2]. Meningioma classification and treatment have evolved over the past eight decades. Since Bailey, Cushing, and Eisenhart’s description of meningiomas in the 1920s and 1930s [3,4,5], there have been continual advances in clinical stratification by histopathology, radiography and, most recently, molecular profiling, to improve prognostication and predict response to therapy. Precise and accurate classification is essential to optimizing management for patients with meningioma, which involves surveillance imaging, surgery, primary or adjuvant radiotherapy, and consideration for clinical trials. This process is challenging, given outcomes that range from an indolent tumor that never grows to aggressive meningiomas that cause mortality. Currently, the World Health Organization (WHO) grade, extent of resection (EOR), and patient characteristics are used to guide management. While these have demonstrated reliability, a substantial number of seemingly benign lesions recur, suggesting opportunities for improvement of risk stratification. Furthermore, the role of adjuvant radiotherapy for grade 1 and 2 meningioma remains controversial, with debate about whether to radiate subtotally resected WHO grade 1 tumors or gross totally resected WHO grade 2 tumors [6,7]. Over the last decade, numerous studies investigating the molecular drivers of clinical aggressiveness have been reported, with the identification of key markers that may carry clinical utility (Figure 1). We review the historical context of current practices (Figure 2), highlight recent molecular discoveries, and discuss the challenges of translating these findings into clinical practice.

## 2. Current Clinical and Pathological Classification with Historical Context

### 2.1. Clinical Features

Several preoperative patient factors predict WHO grade and recurrence. Multiple studies, including one with 1113 patients, found that WHO grade 2 tumors were more likely to be associated with tumor size >3.2 cm, a non-skull base location, and male gender [8,9,10,11,12,13]. Young age is associated with shorter PFS [14]. Finally, malignant meningiomas are more common in African Americans [15]. Machine learning algorithms have shown that patient demographics, radiographic features, and radiomic features such as sphericity have similar accuracy in predicting PFS and overall survival as models based on meningioma grade and EOR [16]. As standardization, generalizability, and reproducibility improve, radiomics may play a role in the evaluation and management of meningioma [17].

### 2.2. Simpson Grade

The importance of EOR in decreasing recurrence has been foundational in meningioma care since the Simpson grade was described in 1957 [18]. The scale is graded I–V, with a grade I resection being complete removal of the tumor, the involved dura, and the involved bone; grade II being complete removal of the tumor and coagulation of the dura; grade III being complete removal of the tumor without coagulating the dura; grade IV being subtotal removal; and grade V being a biopsy or simple decompression. The scale is significantly associated with five-year recurrence [19,20]. A Simpson grade 0, defined as resection of a 2 cm margin of dura around the tumor, which may reduce the rate of recurrence, is often impractical anatomically [21]. Likewise, the ability to achieve a Simpson 1 resection is often limited by anatomical considerations, such as involvement of a patent’s superior sagittal sinus.

The ability to perform safe and complete resections has improved greatly since the 1950s, based on improvements in imaging, instrumentation, intraoperative navigation, and the operative microscope. Thus, the relevance of the Simpson grade is debated [22]. Several studies failed to show a significant difference in patient recurrence between Simpson grade I, II, and III resections [23,24,25,26,27]. However, Simpson grade IV and V resections are still associated with shorter PFS [28]. These findings are likely related to improvements in microsurgical technique, resulting in small tumor volume with subtotal resection [22]. Many surgeons continue to use Simpson grade to communicate EOR, as complete safe resection remains the operative goal.

### 2.3. WHO Grade

The first WHO classification (1979) categorized meningiomas based on histopathological architecture into meningothelial, fibrous, transitional, psammomatous, angiomatous, hemangioblastic, hemangiopericytotic, and papillary subtypes, or as anaplastic, which behaved aggressively [29]. Meningiomas were defined as extra-parenchymal tumors that could be cured surgically [30]. In 1993, the WHO classification added atypical meningiomas, defined as tumors with brisk mitotic activity (4+ mitotic figures per 10 HPF) and hypercellularity without frank anaplasia [31]. Notably, counting mitosis per 10 HPF has more than a 20% discordance between observers [32]. The 2000 WHO classification added clear cell and chordoid histology and brain invasion as a criteria for the atypical grade [33,34,35]. In 2007, additional histopathological criteria (three or more of spontaneous necrosis, sheeting, high cellularity, high nuclear-to-cytoplasmic ratio, and prominent nucleoli) were added to classify a meningioma as grade 2 [36]. Brain invasion alone became sufficient for the diagnosis of atypical meningioma in the 2016 WHO classification [36,37].

The current 2021 WHO classification categorizes meningiomas into three grades with 15 histopathologic subtypes [38]. Nine variants are grade 1, while clear cell and chordoid histology are grade 2. Rhabdoid and papillary histology no longer are criteria for grade 3. The majority of meningiomas are classified as WHO grade 1 and have an 86% 5-year progression-free survival (PFS) regardless of EOR and 96% 5-year PFS after gross total resection (GTR) [37,39,40,41]. Nevertheless, long-term studies show up to 38% of gross totally resected grade 1 meningiomas eventually recur, suggesting that WHO grade alone is not sufficient to predict clinical outcomes [19,39,42,43]. WHO grade 2 (atypical) and 3 (anaplastic) meningiomas have a more aggressive clinical course, with recurrence rates of 20–70% at 5 years, despite surgical resection and adjuvant radiotherapy [37,44]. Survival is shorter for high-grade meningiomas, with a median of 80% of WHO grade 2 and 40% of WHO grade 3 patients surviving 5 years after diagnosis [45]. Finally, molecular changes have been added to the WHO diagnostic criteria, with TERT promoter mutations and homozygous CDKN2A/B loss being sufficient for grade 3 classification. The WHO classification remains the foundation of pathological diagnosis; however, limitations remain due to interobserver variability in histologic analysis, potential under-sampling due to intratumor heterogeneity, and questionable reliability of brain invasion to predict recurrence [32,46,47,48].

### 2.4. Proliferation Index

The proliferation index (MIB-1 index or Ki-67 antigen) is a continuous variable that reports the percentage of cells dividing within a tumor. Despite multiple studies, there is no clear-cut point to define the threshold that independently predicts meningioma recurrence [49,50,51]. Interobserver and institutional differences in evaluating and reporting the MIB-1 index and intratumoral heterogeneity, with hotspots of high Ki-67 activity and areas of low activity, limit generalizability. In particular, there can be differences in the staining methodology; in particular, a lack of accurate quantification and relying on an estimate of the percent staining creates significant interobserver variability. Various studies have suggested MIB-1 cut-off points of 3%, 5%, or 10% as clinically meaningful based on an association with increased risk of progression or recurrence [26,40,46,52]. Notably, the MIB-1 index carries greater prognostic utility when considered in conjunction with histopathology and EOR. One study found that for WHO grade 1 meningiomas undergoing Simpson grade II or III resection, an MIB-1 value of 3% or higher was associated with greater recurrence [26]. Another study of 239 WHO grade I meningiomas found that after GTR, when the MIB-1 index was >4.5%, the recurrence rate was the same (18%) as after subtotal resection (STR) [53]. Among atypical meningiomas after GTR, adjuvant radiation therapy (RT) was associated with a longer PFS when the MIB-1 index was >7% [44]. Thus, the MIB-1 index may be useful in refining predictions of post-operative recurrence when considered with additional factors, but its role as a sole prognostic variable remains unclear. Technical advances, including automated quantification, may improve reproducibility and allow integration of MIB-1 into generalizable prognostic criteria for meningioma [54].

## 3. Recent Advances and Current Evidence for Molecular-Based Classification

Contemporary management of meningiomas depends entirely on histologic, radiographic, and surgical (EOR) data. Despite progressive improvements in these modalities, many patients have recurrences that are unexpected based on benign histopathology, while others do not recur despite having aggressive histopathology. The challenges for clinicians to reliably classify patients into risk groups suggest room for improvement in our current methods. Over the last two decades, scientific advances in high-throughput sequencing have led to an exponential rise in molecular research on meningiomas. Though the clinical significance of these results has not been validated in prospective studies due to the long natural history of meningioma, they are increasingly informing prognostication [55,56]. Analogous discoveries in glioma and other forms of central nervous system (CNS) neoplasia have led to dramatic re-classifications of these lesions, with recognition that the underlying biology is the principal determinate of clinical course. Next, we review and summarize published molecular findings, with a focus on well-validated studies with translational potential.

### 3.1. Chromosomal Copy Number Variants

Meningiomas have been extensively studied with karyotyping and cytogenetics, beginning in the 1970s [57,58,59,60,61]. Most sporadic meningiomas exhibit inactivation of the NF2 tumor suppressor gene, which is lost via copy number variants and/or somatic damaging mutations in 60–80% of cases [62,63,64,65]. NF2 inactivation is associated with atypia and enriched in higher-grade meningiomas [66]. TIMP3 is another gene located near NF2 on chromosome 22 that has tumor suppressor-like properties in addition to inhibiting matrix metalloproteinases, and can be hypermethylated in meningiomas. This hypermethylation is associated with more aggressive high-risk meningiomas and may play a role in malignant conversion of meningiomas [67,68]. High-grade meningiomas often have frequent chromosomal aberrations, such as losses of 1p, 9p (CDKN2A/B), 10q, and 14q, and/or gains of 1q [69,70,71,72,73]. The number of chromosomal aberrations typically increases with histopathologic grade [43,70,74,75,76,77,78,79,80]. Loss of chromosomes 1p and 14q are the second and third most common chromosomal changes, respectively, and are associated with higher-grade meningiomas and reduced PFS [81,82,83,84,85]. Other events have been documented at a lower frequency, including loss of chromosomes 10, 6q, 18q, and sex chromosomes and gains of chromosomes 9q, 12q, 15q, 17q, and 20q [81,86]. Deletions of chr9p21, which includes the tumor suppressor genes CDKN2A (p16) and CDKN2B, are associated with anaplastic meningiomas, poor prognosis, and aggressive clinical behavior [86,87,88,89,90,91].

There have been several proposed methods for meningioma risk stratification and prediction of recurrence using karyotyping and cytogenetics [14,43,74,89,92,93,94]; however, the ability of copy number variation (CNV) to predict clinical behavior is still debated. Complex karyotypes are associated with worsened outcomes [14,43,74,83], though the sensitivity for predicting aggressiveness is often limited. The inclusion of additional clinical features, such as gender, age, tumor size, and location may improve prognostic value. Muti-factorial assessments, including mitotic count, CDKN2A/B status, and CNVs, can differentiate between aggressive WHO grade 1 and well-behaved WHO grade 2 meningiomas, which is useful for risk stratification [86]. Meningioma cytogenetics are not yet included in the WHO guidelines, though loss of chromosome 1p, 6, 9p, and 14, or gain of 1q, should trigger clinical concern for aggressive course and consideration of adjuvant radiotherapy [86,95].

### 3.2. Somatic Mutations

Multiple large-cohort sequencing studies have established the landscape of somatic mutations in meningiomas, identifying mutually exclusive genomic subgroups that account for over 80% of all cases [9,66,96,97]. Bi-allelic loss of the tumor suppressor NF2 is associated with approximately one-half of sporadic meningiomas and most syndromic cases. These alterations can co-occur with mutations in SMARCB1, which also resides on chromosome 22 near the NF2 gene, with meningiomas typically found near the midline falx [98]. Among non-NF2 cases, activating mutations in the oncogenic PI3K (AKT1, PIK3CA) and Hedgehog (SMO) pathways are found primarily in the skull base [66,96]. Other variants in genes previously unreported in cancer have also described, such as TNF receptor-associated factor 7 (TRAF7), Krupple-like factor 4 (KLF4), and RNA-polymerase II (POLR2A) [66]. Meningiomas with KLF4 or PI3K pathway variants nearly always harbor a concomitant mutation in TRAF7, which occurs in the WD40-repeat region of TRAF7. KLF4 is one of four Yamanaka factors that can induce pluripotency in somatic cells [99], while POLR2A encodes the enzyme responsible for transcription of eukaryotic messenger RNA [9]. The oncogenic mechanisms associated with these variants are under investigation.

The reported mutations stratify into mutually exclusive genomic groups that exhibit correlations with molecular, pathological, and clinical features, including location, histology, and WHO grade [9,100,101]. Non-NF2 meningiomas are found along the skull base [9,101,102], with those harboring Hedgehog activating events located in the ventral midline [66,96,103]. By contrast, NF2-mutated meningiomas are located along the convexity and falx and are often the transitional/fibroblastic subtype [104,105]. Secretory meningiomas, a rare subtype associated with peritumoral edema, uniformly harbor co-mutation of TRAF7/KLF4 [66,106,107]. The association of mutations with clinical features suggests different underlying oncogenic mechanisms or cells of origin.

Somatic variants are currently not evaluated in the routine management of meningiomas, but they may predict clinical course [103,108,109]. Mutations in the promotor region of *Telomerase Reverse Transcriptase* (*TERTp*) are the one exception and are associated with malignant progression and an independent criterion for WHO grade 3 diagnosis [110,111,112,113]. Activating mutations in the PI3K pathway are predictive of shorter time to recurrence [108,109], while meningiomas in the hedgehog subgroup exhibit an overall increased rate of recurrence [103,108]. Variants in *SMARCB1* and *SMARCE1*, components of the SWI/SNF chromatin remodeling complex, are enriched in higher-grade meningiomas [56,98,101], and *BAP1* alterations are associated with rhabdoid morphology [114], though the predictive ability of these events is not established. Prospective studies that stratify patients according to *TERTp* and other somatic mutations will be essential before widespread clinical adoption.

### 3.3. Epigenetic and Transcriptional Classification

The role of epigenetic profiling in clinical practice remains an emerging area, which includes assays measuring DNA methylation, H3K27ac ChIP-seq, and other non-coding alterations. In meningiomas, several studies have reported associations between epigenetic alterations and outcome [95,100,115,116], defining associated molecular pathways and transcriptional changes that could underlie tumor aggressiveness. DNA methylation profiling is increasingly being used to define high-risk molecular groups, though availability of testing remains a barrier at many hospitals and institutions [117,118].

Several methylation classification schemes have been proposed using genome-wide clustering approaches [28,95,100,116,119,120]. Sahm et al. described unsupervised clustering of 497 meningiomas into three major epigenetic groups, with subdivision into six subgroups that correlated with clinical outcome [28]. Maas et al. built on this system and combined the Sahm methylation class with WHO grade and CNVs in 1p, 6q and 14q to develop an integrated score to predict outcome, but this still requires methylation profiling [118]. Furthermore, they proposed a schema to guide molecular workup, which is useful when working with limited resources. By clustering multiple molecular profiling approaches, including methylation, Nassiri et al. proposed four methylation subgroups with distinct clinical outcomes [116]. Choudhury et al. profiled 565 meningiomas with long-term clinical follow-up and identified three methylation classes—Merlin-intact, Immune-enriched, and Hypermitotic—and identified biological mechanisms distinct to each subgroup [95]. The two high-risk groups proposed by Nassiri et al. are subgroups within the Hypermitotic methylation group [121]. The Choudhury methylation groups predicted PFS with greater accuracy than the WHO grade and proposed inhibition of CDK4/6 as a rational treatment for Hypermitotic meningiomas. The results and post hoc analysis of Alliance A071401, which has an arm testing Abemaciclib that has completed accrual, are pending. Challenges to the clinical adoption of DNA methylation profiling include availability and cost, as well as the labor and variability in data analysis and post-processing pipelines.

Transcriptional studies have also identified key pathways associated with meningioma development and progression, as well as insights into features associated with poor clinical outcomes. Several studies have identified gene expression signatures that are predictive of clinical course, including WHO grade, recurrence, and mortality [122,123,124]. Recently, Chen et al. reported a 34-gene panel that independently predicted recurrence with a higher area under the curve (AUC) than the WHO grade or any of the proposed CNV-based grading schemes [125]. Furthermore, this gene panel was the first biomarker to predict radiotherapy responses and was validated across 12 sites and more than 1800 meningiomas. In the RTOG-0539 meningioma samples, the gene expression panel would have refined management, either to give radiation, or to withhold it, in nearly 30% of the patients. As this panel becomes prospectively validated and commercially available (currently a research tool), it should help refine care for patients.

The role of specific genes or gene expression pathways has also been investigated, with the hypothesis that select mediators may drive progression or therapeutic resistance. *FOXM1*, one of the earliest transcription factors found to be overexpressed in meningiomas [126], has emerged as a candidate driver of aggressive phenotypes. Expression of this gene is elevated in high-grade meningiomas and is associated with decreased PFS and overall survival [115]. Interestingly, the *FOXM1* and *E2F* transcriptional pathways have been found to underlie de novo formation of atypical meningiomas, while progressing meningiomas harbor *TERT* promotor mutations [100]. The downstream mechanisms associated with *FOXM1* activation are yet to be fully elucidated; however, a *FOXM1*/*WNT* signaling axis that drives proliferative activity has recently been proposed [115]. Consistent with this hypothesis, previous studies have identified increased *WNT* pathway expression among higher-grade meningiomas [127].

### 3.4. PET Imaging and Radiomics

Somatostatin receptor 2A (SSTR2A)-based PET imaging, such as DOTATATE-PET, is emerging as a powerful tool to guide meningioma care and is useful in defining radiotherapy targets as well as differentiating recurrence from scar tissue [128,129,130]. It can highlight intraosseous extension of tumors and can improve radiotherapy control when used to define radiation target volumes [131,132]. There are case reports and on-going trials of DOTATATE coupled to Lutitium-77 as a theranostic treatment strategy [133].

Analyses of MRI imaging data have been extensively reported in a search for prognostic radiographic features that predict grade, invasion, recurrence, and survival [134,135,136,137,138,139]. These studies use various methods of machine learning, including random forests, support vector machines, and convolutional neural networks, to extract non-discrete imaging features related to lesion shape and texture. The most significant features are then used to predict clinical parameters and prognostic features. Clinically significant radiomic features have been reported in the literature to specifically include gray-level co-occurrence and gray-level non-uniformity (cluster prominence) across T1, T2, and fluid-attenuated inversion recovery (FLAIR) sequences and have been predictive in tumor grading [134,138,139,140]. These features have been combined with radiologic parameters, such as cystic components, to have a greater prediction of high-risk atypical meningiomas [139,141]. Additional radiologic features that correspond to higher grade, local recurrence, and poor overall survival include apparent diffusion coefficient (ADC) hypointensity, peritumoral edema, irregular shape, and heterogeneous enhancement [136]. The utility of radiomic approaches, however, is markedly limited by the challenges in generalizing this technology into a clinical workflow.

## 4. Conclusions

**Figure 3:**
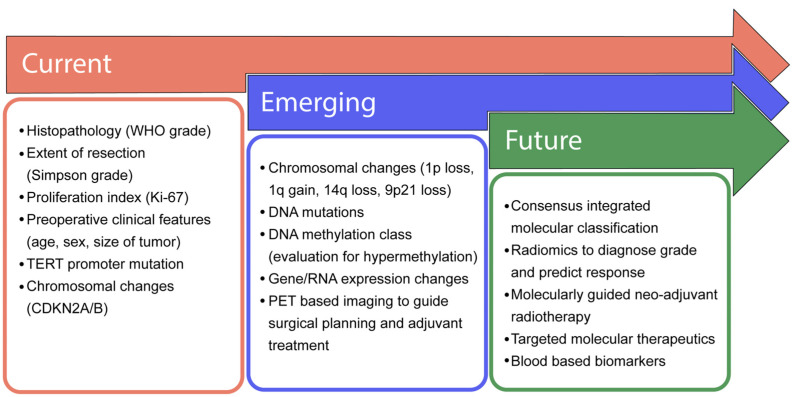
**Progressive advancement in clinical management of meningiomas**. In most clinical settings, the current management of meningiomas is determined by histopathological markers and the extent of surgical resection. Emerging diagnostics include the consideration of copy number events and advanced molecular profiling. As our understanding of meningioma biology matures, the emergence of consensus-integrated paradigms will guide the selection of adjuvant therapies and the need for more frequent follow-up and imaging.

**Figure 4:**
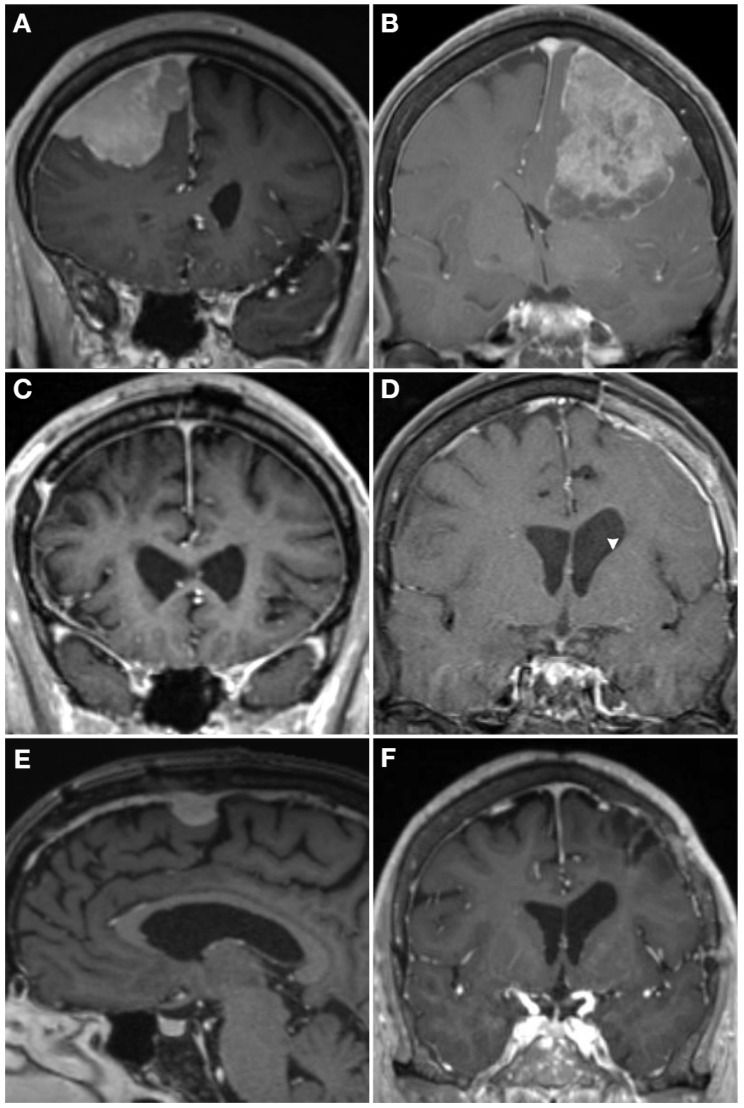
**Case examples demonstrating how molecular profiling can inform care**. T1 post-contrast MRI from two WHO grade 2 meningiomas (**A**,**B**) who both presented with a seizure and underwent gross total resection (**C**,**D**). The patient on the left had a hypermitotic methylation profile and chromosome losses at 1p, 6, 14, haplo-insufficiency of CDKN2A, and 22q, making it a Driver et al. integrated grade 3 tumor, with a high Chen et al. gene expression risk score. He underwent 59.4 Gy adjuvant radiotherapy and had an in-field recurrence (**E**) at 15 months post-operative. The patient on the right also had a WHO grade 2 meningioma that had an immune-enriched methylation profile and chromosome 8 loss with haplo-insufficiency of chromosome 22, making it a Driver et al. integrated grade 1 tumor [86], and had a low Chen et al. gene expression risk score [126]. She was observed given the favorable molecular profile without adjuvant radiotherapy and had no recurrence at 2 years post-operative (**F**).

Molecular characterization of meningioma has progressed rapidly, and insights revealed by these studies are being translated into clinical practice. Emerging molecular data are increasingly being incorporated into clinical practice, including high-risk copy number variants, especially the loss of 1p in combination with 22q loss, as well as CDKN2A/B loss or hypermethylation and TERT promoter mutation (Figure 1 and Figure 3). Emerging data support evaluation of these variants to assess recurrence risk and trigger consideration of adjuvant radiotherapy. How DNA methylation profiling and gene expression-based profiling will be incorporated into clinical practice will be determined in the coming years, something that is currently under discussion by the c-IMPACT NOW working group, and offers the potential to refine care for a significant number of patients. Emerging molecular markers are already being used clinically to inform care (Figure 4) but will require prospective validation. By understanding the molecular pathology driving meningioma growth, we anticipate novel targeted treatments can be developed. At present, molecularly stratified clinical trials are beginning to bring precision medicine to meningioma (Alliance A071401). In the future, we anticipate a consensus molecular classification of meningioma, as well as radiomic and blood-based biomarkers to predict tumor grade and inform clinical management non-invasively. The future is exciting, as the bench-to-bedside cycle continues for meningioma, increasing our understanding of the biology of the disease and using those insights to inform management and develop novel therapeutics.
